# Occurrence and Multi-Locus Analysis of *Giardia duodenalis* in Coypus (*Myocastor coypus*) in China

**DOI:** 10.3390/pathogens10020179

**Published:** 2021-02-07

**Authors:** Zhaohui Cui, Deguo Wang, Wen Wang, Ying Zhang, Bo Jing, Chunyan Xu, Yuanchai Chen, Meng Qi, Longxian Zhang

**Affiliations:** 1 Key Laboratory of Biomarker Based Rapid-Detection Technology for Food Safety of Henan Province, Food and Pharmacy College, Xuchang University, Xuchang 461000, China; czh9008@xcu.edu.cn (Z.C.); wangdg666@126.com (D.W.); 2 College of Animal Science, Tarim University, Alar 843300, China; 18699708588@163.com (W.W.); yingzhang782@gmail.com (Y.Z.); 120050010@taru.edu.cn (B.J.); 120170015@taru.edu.cn (C.X.); 3 College of Veterinary Medicine, Henan Agricultural University, Zhengzhou 450046, China; chenyc217@gmail.com

**Keywords:** *Giardia duodenalis*, coypus (*Myocastor coypus*), multi-locus genotype, genetic variation, zoonotic genotypes, PCR (polymerase chain reaction), China

## Abstract

*Giardia duodenalis* is a major gastrointestinal parasite found globally in both humans and animals. This work examined the occurrence of *G. duodenalis* in coypus (*Myocastor coypus*) in China. Multi-locus analysis was conducted to evaluate the level of genetic variation and the potential zoonotic role of the isolates. In total, 308 fecal samples were collected from seven farms in China and subjected to PCR screening to reveal *G. duodenalis*. Notably, *G. duodenalis* was detected in 38 (12.3%) specimens from assemblages A (*n* = 2) and B (*n* = 36). Positive samples were further characterized by PCR and nucleotide sequencing of the triose phosphate isomerase (*tpi*), beta giardin (*bg*), and glutamate dehydrogenase (*gdh*) genes. Multi-locus genotyping yielded 10 novel multi-locus genotypes (MLGs) (one MLG and nine MLGs for assemblages A and B, respectively). Based on the generated phylogenetic tree, AI–novel 1 clustered more closely with MLG AI-2. Furthermore, within the assemblage B phylogenetic analysis, the novel assemblage B MLGs were identified as BIV and clustered in the MLG BIV branch. This is the first report of *G. duodenalis* in coypus in China. The presence of zoonotic genotypes and subtypes of *G. duodenalis* in coypus suggests that these animals can transmit human giardiasis.

## 1. Introduction

*Giardia duodenalis* (syn. *G. lamblia*, *G. intestinalis*) is a flagellate protozoan parasite recognized as a significant global contributor to diarrheal disease, affecting humans, domestic animals, and wildlife across the globe [1,2]. The majority of *G. duodenalis* infections are asymptomatic; however, in rare cases, some patients may experience severe gastrointestinal disturbances for several weeks [3]. As *G. duodenalis* utilizes the fecal–oral route for lifecycle maintenance, projections indicate that this parasite causes ~28.2 million foodborne disease cases [4,5]. Based on the above data, the United Nations Food and Agriculture Organization (FAO) and the World Health Organization (WHO) in 2014 ranked *Giardia* 11th of 24 food-borne parasites [4].

The current wide use of genotyping tools has immensely improved our understanding of *G. duodenalis* transmission in humans and animals [5,6]. At least eight genotypes or assemblages have been described, including assemblages A and B containing zoonotic isolates potentially infecting humans and animals, and assemblages C–H, which exhibit specificity to particular animal hosts [7]. Moreover, several molecular markers (triosephosphate isomerase, *tpi*; glutamate dehydrogenase, *gdh* and beta giardin, *bg*) have been developed to create a multi-locus genotyping (MLG) tool for subtyping assemblages A, B, and E and to explore the population genetic characterizations of *G. duodenalis* [6,8]. Subsequently, the MLG tool subdivided assemblage A into sub-assemblages AI, AII, and AIII and assemblage B into sub-assemblages BIII and BIV [9].

In recent years, studies on the epidemiology of *G. duodenalis* have been conducted for humans, non-human primates, ruminants, companion animals, domestic animals, wildlife, and in the environment in China [10]. However, limited information has been provided on the infection rate and genotype characteristics in rodents in China. The coypus (*Myocastor coypus*) is a large, amphibious rodent native to South America, which has become invasive in Europe and other parts of the world except Oceania and Antarctica [11]. Coypus were first introduced to China in 1956, then later widely reared in farms as important fur-bearing animals. The climate of China is very suitable for the growth of coypus, and 16 color-type strains have been bred. The number of the national stock reached more than 400,000 in 2000. To date, little is known about the genetic characteristics of *G. duodenalis* in coypus globally. Only two studies in Italy and the USA reported *Giardia* spp. prevalence in coypus, but neither identified the species [12,13]. Thus, the present study aimed to explore the distribution and genetic diversity of *G. duodenalis* in coypus in China and assess its zoonotic potential based on MLG analysis.

## 2. Results

### 2.1. Occurrence of G. duodenalis

All samples were initially tested using nested PCR amplification of the small subunit ribosomal RNA (SSU rRNA) gene. Of the 308 samples, *G. duodenalis* was present in 38 samples (12.3%, 95% Cl: 8.5–16.2%) (Table 1). Each examined farm had infected animals. Notably, the highest infection rate of *G. duodenalis* in coypus was detected in Baoding (28.6%, 10/35), followed by Ganzhou (25.7%, 9/35), Chengdu (15.0%, 6/40), Laibin (13.6%, 3/22), Yongzhou (13.0%, 3/23), Kaifeng (11.5%, 6/52), and Anyang (1.0%, 1/101) (Table 1).

By age, the highest infection rate was reported in the 3–6-month-old group (14.9%, 7/47), followed by >6-month-old group (14.8%, 29/196), and <3-month-old group (3.1%, 2/65) (Table 2). Furthermore, the correlation of age with the infection rates was evaluated based on the calculated ORs and 95% CI values (Table 2). There was a strong positive correlation between the infection rate and age, with an OR of 5.51 (95% CI: 1.09–27.87, *p* = 0.023) associated with the 3–6-month-old group and an OR of 5.47 (95% CI: 1.27–23.59, *p* = 0.011) associated with the >6-month-old group.

### 2.2. Assemblage A and B Subtypes

Here, two genotypes, assemblages A (2) and B (35), were identified based on sequence analysis of the SSU rRNA, *tpi*, *gdh*, and *bg* loci (Table 1). Notably, assemblage B was the dominant genotype (92.1%, 35/38). Mixed infection was found in one sample. To reveal the genetic diversity of the *G. duodenalis*-positive samples, we sequenced the *tpi*, *gdh*, and *bg* genes, from which 25, 11, and 12 sequences were obtained, respectively (Table 3). 

Of the *tpi* sequences, 3 and 22 belonged to assemblages A and B, respectively. Sequences from all isolates from assemblage A exhibited two single-nucleotide polymorphisms (G155T and G186A) relative to the MN174855 sequence. Within the assemblage B isolates, three subtypes were formed, which were designated as B1 (*n* = 17), B2 (*n* = 3), and B3 (*n* = 2) for convenience. Of note, B1, B2, and B3 sequences were identical to MH644772, KM977653, and HM140711, respectively. At the *gdh* locus, 1 and 10 samples were found positive and identified as assemblages A and B, respectively. The one assemblage A sequence was identical to MN174853. Among the assemblage B sequences, B1 (*n* = 5), B2 (*n* = 3), and B3 (*n* = 2) sequences were identical to KM977648, MK952603, and MK982476, respectively. Sequence analysis demonstrated high genetic diversity in assemblage B at the *bg* locus. Ultimately, five subtypes (B1–B5) were formed in the 11 assemblage B sequences. The subtypes B1 (*n* = 4), B2 (*n* = 2), B3 (*n* = 3), B4 (*n* = 1), and B5 (*n* = 1) exhibited consistency with MT487587, MK982544, MN174847, MT487587, and KY696837, respectively. Moreover, one assemblage A sequence was identical to MN704938.

### 2.3. MLG and Phylogenetic Analysis

**Figure 1:**
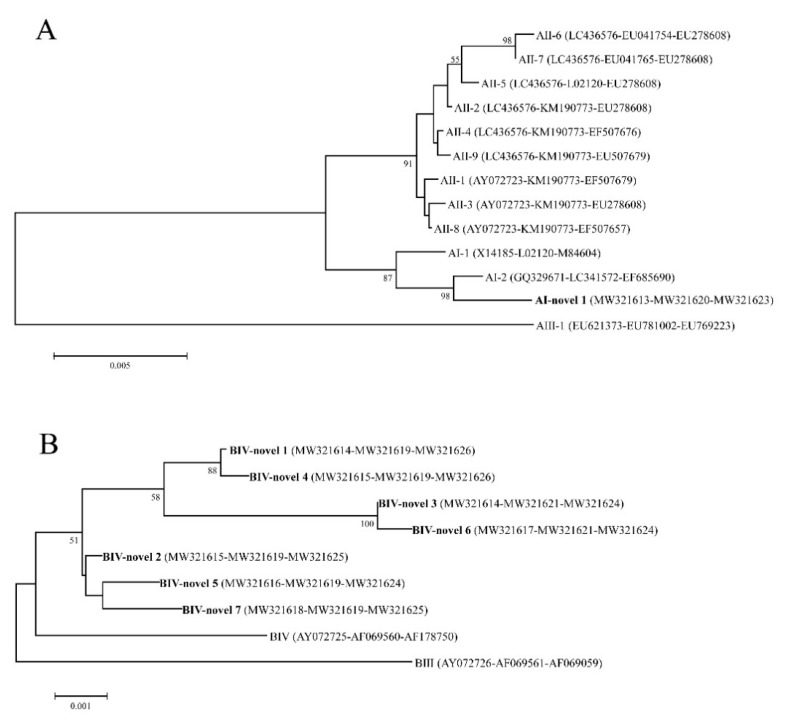
Nucleotide neighbor-joining trees based on concatenated datasets for *bg*, *tpi*, and *gdh* gene sequences of *G. duodenalis* assemblage (**A**,**B**) isolates obtained in this study and sequences retrieved from the GenBank database. Bootstrap values greater than 50% from 1000 replicates are shown on nodes. The bold texts represent the isolates of this study.

Collectively, sequence data sets from the three loci were available from 10 isolates. Multi-locus genotyping yielded 10 novel MLGs (one MLG for assemblage A and nine MLGs for assemblage B) (Table 3). The single MLG in assemblage A was identified as AI–novel 1, whereas the nine MLGs in assemblage B were designated from BIV–novel 1 to BIV–novel 7. Phylogenetic relationships of MLGs in assemblages A and B with the reference genotypes are illustrated in Figure 1. Based on the phylogenetic tree, AI–novel 1 identified in the present study clustered more closely to MLG AI-2 (Figure 1A). Within the assemblage B phylogenetic analysis, all MLGs in assemblage B were identified as BIV and clustered in the MLG BIV branch (Figure 1B).

## 3. Discussion

*G. duodenalis* is very commonly found in humans and domestic animals, as revealed by numerous prevalence studies across the globe [7,10,14]. The molecular epidemiology of *G. duodenalis* has been widely studied in livestock, which revealed its transmission dynamics and zoonotic significance in these animals. However, the parasite has not been extensively investigated in rodents; therefore, very little knowledge on the distribution, genetic diversity, and zoonotic potential of *Giardia* spp. in these animal hosts has been published [6,7,15,16]. Reports have demonstrated that *G. duodenalis* infections in rodents in Australia, Belgium, China, Croatia, Germany, Malaysia, Poland, Romania, Spain, Sweden, and the USA (Table 4) have prevalence rates ranging from 1.2% to 100% [12,13,14,15,16,17,18,19,20,21,22,23,24,25,26,27,28,29,30,31,32,33,34]. Herein, molecular analysis of 308 fecal samples collected from coypus in six provinces in China confirmed a *G. duodenalis* prevalence of 12.3% (38/308). To the best of our knowledge, this is the first molecular study of *G. duodenalis* infections in coypus, except for two studies in Italy and the USA that assessed *Giardia* spp. infection in coypus with the prevalence of 0% (0/153) and 73.3% (22/30), respectively, using an immunoenzymatic assay [12,13].

Based on the current knowledge, eight *Giardia* species are considered valid, including *G. duodenalis*, *Giardia agilis*, *Giardia ardeae*, *Giardia psittaci*, *Giardia muris*, *Giardia microti*, *Giardia peramelis*, and *Giardia cricetidarum* [4,7]. In particular, two host-adapted species of *Giardia* spp. have been detected in rodents, including *G. muris* and *G. microti* [20,35]. To date, in China, *G. duodenalis* has been reported in rodents of several genera, including Chinchillas (*Chinchilla lanigera*), Asian house rats (*Rattus tanezumi*), brown rats (*Rattus norvegicus*), house mice (*Mus musculus*), chipmunks (*Eutamias asiaticus*), and bamboo rats (*Rhizomys sinensis*) [18,19,26,30]. In previous work, seven assemblages of *G. duodenalis* were found in rodents, including A, B, C, D, E, F, and G (Table 4). Both zoonotic and rodent-specific assemblages A, B, and G of *G. duodenalis* have been detected in rodents in China. Herein, the assemblages A and B were identified; assemblage B was the predominant genotype in coypus. It is worth noting that *G. duodenalis* assemblages A and B are important human pathogens; of them, assemblage B is more commonly reported in Asia, Oceania, Europe, and Africa than assemblage A [5,15,36]. We also reported mixed infections in the present study. Of note, the use of assemblage-specific primers in addition to MLST data is advocated in molecular epidemiological surveys, as mixed infections are likely to be underestimated [15,37]. Mixed infections are likely to be one of the main reasons for the adoption of multiple genetic markers in identifying distinct assemblages in the same sample [8,15]. Consequently, further studies on the molecular epidemiology of *G. duodenalis* is warranted to improve our understanding of the genetic diversity in rodents in China.

In recent studies, the MLG tool based on sequence analysis of the *gdh*, *bg*, and *tpi* loci represents a more informative approach for genotyping and elucidating the characteristics of *G. duodenalis* in different hosts, in addition to its zoonotic potential [9,15,19]. This has permitted the identification of sub-assemblages within assemblage A and B: AI to AIV and BI to BIV. Moreover, there are differences in the distribution of these sub-assemblages among hosts; for instance, human *Giardia* isolates mainly belong to sub-assemblage AII but also AI, whereas animals harbor AI, AII, and AIII [15]. In this study, 10 *G. duodenalis* isolates from coypus were successfully sequenced at all three gene loci; the sequence analysis of which resulted in one MLG in assemblage A and nine MLGs in assemblage B. Based on the phylogenetic analysis, the one MLG in assemblage A (AI–novel 1) was clustered with sub-assemblage AI-2, whereas the nine remaining MLGs in assemblage B (BIV–novel 1 to 7) were clustered with sub-assemblage BIV. Sub-assemblage AI was previously reported in various hosts in China, including humans, cattle, goats, sheep, dogs, cats, pigs, chinchillas, and chipmunks [6,10]. Other researchers also found sub-assemblage BIV in humans and non-human primates [6,10]. Additionally, compelling evidence on zoonotic transmission of *G. duodenalis* assemblage AI was revealed by epidemiological data showing a highly significant association between *Giardia* infection of schoolchildren and the presence of *Giardia*-positive dogs in the same household in Mexico [38]. Therefore, further studies into the epidemiology of *G. duodenalis* in handlers/workers on farms should be initiated to address the zoonotic potential of *G. duodenalis* in coypus.

## 4. Materials and Methods

### 4.1. Ethics Statement

This study was performed with strict adherence to the recommendations of the Guide for the Care and Use of Laboratory Animals of the Ministry of Health, China. The research protocol was reviewed and approved by the Research Ethics Committee of Tarim University (approval no. ECTU 2018-0026). Farm owners gave permission before we commenced fecal sample collection.

### 4.2. Study Area and Sample Collection

A total of 308 fecal samples were collected during autumn, winter, and spring of 2018 and 2019 from seven farms in Hebei Province (Baoding), Henan Province (Anyang and Kaifeng), Sichuan Province (Chengdu), Hunan Province (Yongzhou), Jiangxi Province (Ganzhou) and Guangxi Zhuang Autonomous Region (Laibin) in China (Table 1). The breeding scale of coypus was about 500–2000 in each farm, and the breeding conditions of each farm were basically the same. Approximately 10–15% of coypus representing each age group were investigated at each farm. All the fecal samples were collected immediately after excretion and placed in a plastic container; we recorded the date, site, age, and health condition at collection time. All samples were shipped to the laboratory in a cooler with ice packs within 48 h and stored at 4 °C.

### 4.3. DNA Extraction and Nested PCR Analysis

The genomic DNA sample was extracted from approximately 200 mg of each fecal specimen using a commercial E.Z.N.A^®^ Stool DNA Kit (Omega Bio-Tek Inc., Norcross, GA, USA) following the protocol stipulated by the manufacturer. The quantity of nucleic acid in samples was photometrically estimated at OD_260_ and stored at −20 °C for subsequent molecular analysis.

Nested PCR amplification of the SSU rRNA gene was employed to screen *G. duodenalis* infection [39]. The *G. duodenalis*-positive samples were further analyzed via nested PCRs of gene loci *tpi*, *gdh*, and *bg* (see Table S1 and Figure S1) [8,22,40]. Samples positive for all four loci were used to assess the MLGs of *G. duodenalis*.

### 4.4. Sequencing and Phylogenetic Analysis 

Bi-directional sequencing of all the secondary PCR products from SSU rRNA, *tpi*, *gdh*, and *bg* loci was performed at the biotechnology company, GENEWIZ (Suzhou, China). The sequence assembly and editing were performed with the DNAstar Lasergene Editseq 7.1.0. Multiple-sequence alignment analysis was employed on the obtained and GenBank reference sequences using software ClustalX 2.1 to ascertain the genotypes and subtypes of *G. duodenalis*. We concatenated sequences for each positive isolate to obtain a multi-locus sequence (*bg*, *tpi*, *gdh*) in accordance with a previous report [41]. Phylogenetic analyses of the concatenated MLG sequences were achieved using neighbor-joining methods based on the Kimura-2 parameter model in MEGA 7.0.

### 4.5. Statistical Analysis

The differences in infection rates among locations and age groups were compared with the χ2 test performed in SPSS 18. At the level of *p* value < 0.05, the differences were appraised as significant.

### 4.6. Nucleotide Sequence Accession Numbers

Here, the representative nucleotide sequences of this study were deposited in the GenBank database under the following accession numbers: MW322754–MW322755, MW321613–MW321626.

## 5. Conclusions

This work presents the first report of *G. duodenalis* infections in coypus in China based on MLG analysis. Multi-locus genotyping yielded 10 novel MLGs, including one assemblage A MLG (AI–novel 1) and nine assemblage B MLGs (BIV–novel 1 to 7). Of note, the presence of zoonotic assemblages and sub-assemblages of *G. duodenalis* in coypus suggests the potential contribution of these animals to human giardiasis transmission.

## Figures and Tables

**Table 1:** Distribution of *G. duodenalis* assemblages in coypus from different farms in China.

Location	Age (Month)	N/T (%; 95% Cl)	Assemblage (No.)	SSU rRNA (No.)	*tpi* (No.)	*gdh* (No.)	*bg* (No.)
Baoding, Hebei	<3	2/13 (15.4; 0–38.8)	B (2)	B (2)	B (1)	B (1)	B (1)
	3–6	5/10 (50.0; 14.0–85.9)	B (5)	B (5)	B (4)	B (2)	B (3)
	>6	3/12 (25.0; 0–53.7)	B (3)	B (3)	B (1)	B (1)	B (1)
	Subtotal	10/35 (28.6; 12.2–44.9)	B (10)				
Anyang, Henan	<3	0/52					
	3–6	0/10					
	>6	1/39 (2.6; 0–8.8)	B (1)	B (1)			
	Subtotal	1/101 (1.0; 0–3.4)	B (1)				
Kaifeng, Henan	3–6	2/27 (7.4; 0–19.1)	B (2)	B (2)	B (1)		
	>6	4/25 (16.0; 0–32.4)	B (4)	B (4)	B (2)		
	Subtotal	6/52 (11.5; 1.9–21.2)	B (6)				
Chengdu, Sichuan	>6	6/40 (15.0; 2.7–27.3)	B (6)	B (6)	B (6)	B (3)	B (3)
Yongzhou, Hunan	>6	3/23 (13.0; 0–28.9)	B (3)	B (3)	B (1)		
Ganzhou, Jiangxi	>6	9/35 (25.7; 9.8–41.6)	B (9)	B (9)	B (6)	B (3)	B (3)
Laibin, Guangxi	>6	3/22 (13.6; 0–30.2)	A (2), A + B (1)	A (2), B (1)	A (3)	A (1)	A (1)
Total		38/308 (12.3; 8.5–16.2)	A (2), B (35), A + B (1)				

N = number of positives for *G. duodenalis*; No. = number of samples; T = total analyzed samples.

**Table 2:** Distribution of *G. duodenalis* assemblages in coypus of different ages.

Age (Month)	N/T (%; 95% CI)	Assemblage (No.)	*p-*Value	OR (95% CI)
<3	2/65 (3.1; 0–8.0)	B (2)		1
3–6	7/47 (14.9; 3.7–26.1)	B (7)	0.023	5.51 (1.09–27.87)
>6	29/196 (14.8; 9.6–20.0)	A (2), B (26), A + B (1)	0.011	5.47 (1.27–23.59)

N = number of positives for *G. duodenalis*; OR: odds ratio; T = total of analyzed samples.

**Table 3:** Multi-locus characterization of *G. duodenalis* isolates in coypus in China based on *bg*, *gdh* and *tpi* genes.

Isolate Code	*tpi*	*gdh*	*bg*	MLG Type
118	B1 (MW321619)	PN	PN	
126	A * (MW321620)	A5 (MW321623)	A5 (MW321613)	AI-novel 1
127	A *	PN	PN	
128	A *	PN	PN	
147	B1	B3 (MW321626)	B1 (MW321614)	BIV-novel 1
150	B1	PN	B1	
151	B2 (MW321621)	PN	PN	
155, 256	B1	B2 (MW321625)	B2 (MW321615)	BIV-novel 2
157	B2	B1 (MW321624)	B1	BIV-novel 3
171	B1	B3	B2	BIV-novel 4
182, 184	B1	B1	B3 (MW321616)	BIV-novel 5
189	B1	PN	PN	
197	B3 (MW321622)	PN	B3	
210	B3	B1	PN	
217	B1	PN	PN	
225	B1	PN	PN	
233	B2	B1	B4 (MW321617)	BIV-novel 6
245	B1	PN	PN	
248	B1	B2	B5 (MW321618)	BIV-novel 7
249	B1	PN	PN	
283	B1	PN	PN	
287	B1	PN	PN	
302	B1	PN	PN	

* New variants without heterogeneous positions. MLG: multi-locus genotypes; PN: PCR negative.

**Table 4:** *Giardia duodenalis* infection rates and genotypes in rodents worldwide.

Animal	Location	Positive % (N/T)	Assemblage	Sub-Assemblage	Reference
Ash-grey mouse (*Pseudomys albocinereus*)	Australia	-	E	-	[17]
Asian house rats (*Rattus tanezumi*)	China	6.1 (2/33)	G	-	[18]
Bamboo rat (*Rhizomys sinensis*)	China	10.8 (42/480)	B	-	[19]
Bank vole *(Myodes glareolus)*	Germany	1.3 (4/301)	A, B	-	[20]
Bank vole (*Myodes glareolus*)	Poland	58.3 (849/1457)	-	-	[21]
Beaver (*Castor canadensis*)	USA	33.3 (30/100)	-	-	[13]
Beaver (*Castor canadensis*)	USA	-	B	-	[22]
Black rat (*Rattus rattus*)	Spain	36.2 (42/116)	G	GI, GII	[23]
Brown rats (*Rattus norvegicus*)	Malaysia	3.0 (4/134)	B	-	[24]
Brown rats (*Rattus norvegicus*)	China	6.6 (11/168)	G	-	[18]
Bush rat (*Rattus fuscipes*)	Australia	-	F + C	-	[17]
Chinchillas (*Chinchilla lanigera*)	Belgium	27.5 (22/80)	A, B, C, E	AI, AII, BIV	[25]
Chinchillas (*Chinchilla lanigera*)	China	27.1 (38/140)	A, B	AI, BIV	[26]
Chinchillas (*Chinchilla lanigera*)	Germany	61.4 (326/531)	A, B, D	AI, BIV	[27]
Chinchillas (*Chinchilla lanigera*)	Italy	29.8 (31/104)	B, C		[28]
Chinchillas (*Chinchilla lanigera*)	Romania	55.7 (190/341)	B, D, E	BIII, BIV	[29]
Chipmunks (*Eutamias asiaticus*)	China	8.6 (24/279)	A, G	AI, GI, GII	[30]
Common vole (*Microtus arvalis*)	Poland	74.2 (302/407)	-	-	[21]
Coypus (*Myocastor coypus*)	China	12.3 (38/308)	A, B	AI, BIV	This study
Coypus (*Myocastor coypus*)	Italy	0 (0/153)	-	-	[12]
Nutria (*Myocastor coypus*)	USA	73.3 (22/30)	-	-	[13]
Deer mice (*Peromyscus maniculatus*)	USA	25.5 (53/208)	-	-	[31]
Eurasian field mice (*Apodemus* sp.)	Germany	1.2 (1/82)	A	-	[20]
House mice (*Mus musculus*)	China	3.2 (1/31)	G	-	[18]
House mouse (*Mus musculus domesticus*)	Spain	17.6 (29/165)	G	-	[23]
Muskrat (*Ondatra zibethicus*)	Romania	100 (1/1)	C	-	[32]
Muskrat (*Ondatra zibethicus*)	USA	-	B	-	[22]
Norway rat (*Rattus norvegicus*)	Spain	66.7 (2/3)	B	-	[23]
Prevost’s squirrel (*Callosciurus prevosti*)	Croatia	-	B	-	[33]
Patagonian cavy (*Docilchotis patagonum*)	Croatia	-	B	BIV	[33]
Rat	Sweden	-	G	-	[34]
Yellow-necked mouse (*Apodemus flavicollis*)	Poland	24.4 (150/616)	-	-	[21]

N: number of positives for *G. duodenalis*; T: total of analyzed samples; “-” indicates not available.

## Supplementary Materials

The following are available online at https://www.mdpi.com/2076-0817/10/2/179/s1, Table S1 Primer sequences and reaction conditions used in nested PCR amplifications. Figure S1. Genomic map of the positions of *tpi*, *gdh* and *bg* genes.
