# Supplementary material for: Occurrence and Multi-Locus Analysis of Giardia duodenalis in Coypus (Myocastor coypus) in China
Source: Pathogens. 2021 Feb 7;10(2):179. doi: 10.3390/pathogens10020179 (PMC7914982; doi:10.3390/pathogens10020179)
Supplement: Supplementary file 1 [file pathogens-10-00179-s001.pdf]

Supplementary Materials:

Table S1. Primer sequences and reaction conditions used in nested PCR amplifications.

Target Gene	Primer sequences (5′- 3′)	Annealing (°C)	Target size (bp)	Reference
SSU rRNA	Gia2029 (AAGTGTGGTGCAGACGGACTC)	55		
	Gia2150c (CTGCTGCCGTCCTTGGATGT)			
	RH11 (CATCCGGTCGATCCTGCC)	59	292	[13]
	RH4 (AGTCGAACCCTGATTCTCCGCCCAGG)			
tpi	AL3543 (AAATIATGCCTGCTCGTCG)	50		
	AL3546 (CAAACCTTITCCGCAAACC)			
	AL3544 (CCCTTCATCGGIGGTAACTT)	50	530	[8]
	AL3545 (GTGGCCACCACICCCGTGCC)			
gdh	GDH1 (TTCCGTRTYCAGTACAACTC)	50		
	GDH2 (ACCTCGTTCTGRGTGGCGCA)			
	GDH3 (ATGACYGAGCTYCAGAGGCACGT)	50	530	[14]
	GDH4 (GTGGCGCARGGCATGATGCA)			
bg	G7 (AAGCCCGACGACCTCACCCGCAGTGC)	58		
	G759 (GAGGCCGCCCTGGATCTTCGAGACGAC)			
	2005F (GAACGAACGAGATCGAGGTCCG)	55	511	[15]
	2005R (CTCGACGAGCTTCGTGTT)			

Figure S1.

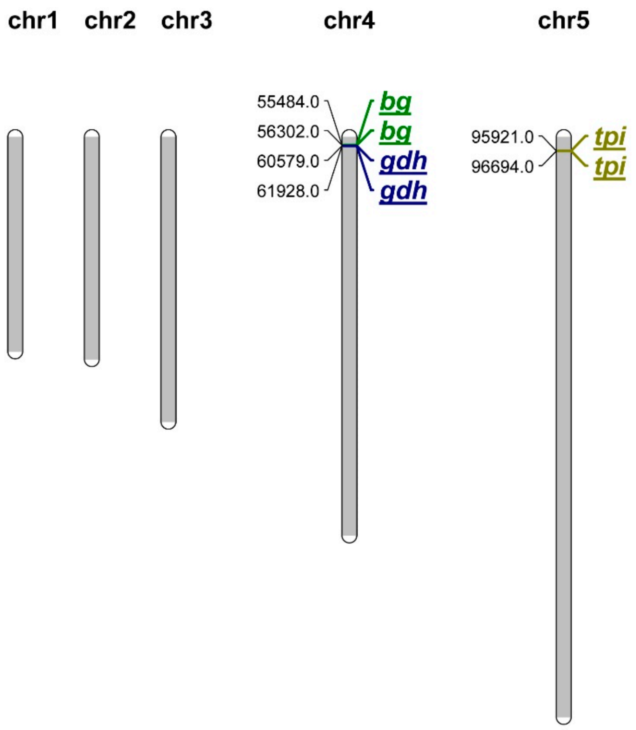

Figure S1. Genomic map of the positions of *tpi*, *gdh* and *bg* genes. The *tpi* gene is at position 95,921–96,694 on the 200-kb long contig ctg02-19, chromosome 5; the *bg* gene is at position 55,484–56,302 on the 90-kb long contig ctg02_35, chromosome 4; the *gdh* gene is at position 60,579–61,928 on the 231-kb long contig ctg02_15, chromosome 4 (data taken from www.giardiadb.org).
